# State-Level Spending on Children Associated with Unequal Benefits to School Readiness

**DOI:** 10.1007/s10995-025-04068-9

**Published:** 2025-02-14

**Authors:** Taryn W. Morrissey, Katherine Engel, Margot I. Jackson

**Affiliations:** 1 School of Public Affairs, American University, Kerwin Hall 4400 Massachusetts Ave NW, Washington, DC 20016 USA; 2 Brown University, Providence, RI USA

**Keywords:** Public spending, School readiness, Disparities

## Abstract

**Objectives:**

To examine associations between state-level public investments in programming for children and parents’ reports of their children’s kindergarten readiness.

**Methods:**

We use regression approaches with publicly available, nationally representative data to examine how time and state variation in public spending on children relates to parents’ concerns about children’s development. We link data on annual state-level spending on health and early learning from the Urban Institute’s State-by-State Spending on Kids Dataset and the National Institute for Early Education Research to child-level data from the 2003/2004, 2007/2008, and 2011/2012 waves of the National Survey of Children’s Health (NCHS), focusing on a subsample of parents with one or more children under age six (*N* = 56,736).

**Results:**

Child-related public spending on both health and early education is associated with decreases in parents’ concerns about their children’s physical health and motor development. A 15% increase in average health spending and early education spending per child per year is associated with a reduction in parents’ concerns about children’s health and motor development of about 3% and 2% of a standard deviation (SD), respectively. Associations between spending and concerns about early learning and social-emotional development are negative but not significant. Among socioeconomically disadvantaged or racial and ethnic minority parents, spending is associated with smaller reductions in concerns.

**Conclusions for Practice:**

Public spending on children is associated with fewer parents’ concerns about their children’s development, but less so among disadvantaged families. It is possible that public spending levels are not adequate to narrow disparities in early opportunity and outcomes.

**Supplementary Information:**

The online version contains supplementary material available at 10.1007/s10995-025-04068-9.

## Significance

What is Already Known on this Subject

What this Study adds

Prior research indicates that state-level public investments in children are positively related to family well-being, but their association with young children’s readiness for kindergarten is unclear.

This study examines how state-level public investments in programming for children and parents’ reports of their children’s kindergarten readiness.

**Supplementary Information:**

The online version contains supplementary material available at 10.1007/s10995-025-04068-9.

## Introduction

Public state-level investments in children, including income supports (Hoynes & Schanzenbach, 2018) investments in housing assistance (Slopen et al., 2018) early childhood and K-12 education spending (Chaudry et al., 2021), and health insurance (Goodman-Bacon, 2016) are strongly and positively related to family well-being and children’s economic opportunity. State-level public spending on programs targeted to children improves their well-being and narrows inequalities in family investments and children’s health and development (Flood et al., 2021; Jackson et al., 2022, 2024). Investments in family resources reduce material hardship, improve household health behaviors, and reduce parenting stress (Collins & Klerman, 2017). Outside of the home, greater public spending on in-kind benefits expands children’s access to resources, including health care settings and schools (Chaudry et al., 2021; Jackson et al., 2016). Overall, income support, health, and educational investments improve the outcomes of low-income children (Jackson, 2015; Markowitz et al., 2017), and public expenditures on families may increase equality of opportunity and reduce socioeconomic (SES) disparities (Bradbury et al., 2015; Corak, 2013; Waldfogel, 2016).

Despite early childhood being a particularly sensitive developmental period (Committee on Integrating the Science of Early Childhood Development Youth, and et al., 2000; Shonkoff, 2010), public spending on very young children is lower and more variable than for children over age five (Greenberg et al., 2021; Lou et al., 2022). The effects of spending on children’s school readiness at kindergarten entry, inclusive of cognitive, social-emotional, and physical development (Duncan et al., 2007), are of particular interest given that school readiness is predictive of longer-term health and well-being and there are large disparities in these outcomes (Duncan et al., 2007; Magnuson & Duncan, 2017), much of which stem from different early environments and opportunities (Bassok et al., 2016; Jackson et al., 2021a, b). These disparities have important downstream effects on educational, health, and economic inequalities (Duncan et al., 2007).

Collectively, state-level investments in in-kind supports may promote higher levels of school readiness by increasing children’s access to resources outside of the home that support their learning and healthy development.Higher per child spending in K-12 and early education is linked to improved educational outcomes, including school readiness (Chaudry et al., 2021). Higher per-enrollee Medicaid spending, for example, is indicative of higher provider reimbursement levels and greater access to providers (Cannon et al., 2018). Likewise, higher per-child spending levels on public preschool, Head Start, and child care may support more children receiving services, smaller class sizes, improved classroom supplies, health services, and increased teacher compensation (Chaudry et al., 2021). In-kind investments, particularly in preschool, may also identify and treat children’s developmental delays, learning or health issues early (Hong et al., 2018), as well as increase parents’ access to information about their children’s development, resulting in greater awareness about potential concerns.

Increased access to out-of-home resources may especially benefit lower-SES and non-White children. SES and racial/ethnic disparities in school readiness persist, notwithstanding some recently evidence of narrowing (Bassok et al., 2019; Magnuson & Duncan, 2017; Reardon & Portilla, 2016). Further, many child-serving public programs, such as Head Start, are means-tested and specifically target lower-SES families. The benefits of these types of targeted government transfers are largest among the most socioeconomically disadvantaged participants (Goodman-Bacon, 2018). Alternatively, the benefits of state-level expenditures may be largest among higher-SES and non-Hispanic White families, given processes of historical and structural racism and classism that perpetuate advantages among these families and contribute to disparities in the health and development (Chaudry et al., 2021; Reardon & Portilla, 2016). Understanding the impact of government expenditures during a highly sensitive period of development is important, given striking disparities in school readiness (Duncan et al., 2007; Reardon & Portilla, 2016) and the lasting impact of early-life circumstances on later youth and adult outcomes (Jackson, 2010).

Using the most recent data available on child-related state spending (Isaacs et al., 2020) linked to child-level data from the waves of the National Survey of Children’s Health (NSCH), we examine the association between state-level spending on children and parental reports about the school readiness of children under six. In addition to considering the aggregate benefits of state-level investments in children, we examine variation by child race, parental educational attainment, and household poverty ratio. We focus on per-child spending on in-kind supports during the early childhood period (prior to elementary school entry), particularly health spending and early childhood education spending.

## Methods

### Data and Sample

Our primary data source is the 2003/2004, 2007/2008, and 2011/2012 waves of the NSCH. This large cross-sectional, nationally and state representative survey assesses the physical and emotional health of children aged 0 to 17 years and factors related to child well-being. In these waves, the NSCH was conducted by the U.S. Department of Health and Human Services.^1^The survey collected data from all 50 states and the District of Columbia from January 2003-July 2004, April 2007-July 2008, and February 2011-June 2012. To collect these data, cross-sectional telephone surveys of parents or guardians with knowledge of the health and health care of the children in the household were completed, resulting in 102,353, 91,642, and 95,677 child-level interviews in 2003/3004, 2007/2008, and 2011/2012, respectively. We use these years of the NSCH because they correspond to the most recent years of state spending available (ending in 2016; described below). Our sample contains responses from parents of children who were under six years of age at the time NSCH interview (*N* = 56,736). Survey weights are included such that these data can be used to generate nationally and state representative estimates.^2^ See the Appendix for details.

### Dependent Variables

Following previous work (Ghandour et al., 2019; Jackson et al., 2021a), we use three measures to assess how state spending is associated with parents’ concerns about their children as indicators of the child’s school readiness: early learning, socio-emotional development, and physical health and motor development. These indicators were adapted from the Parents’ Evaluation of Developmental Status (PEDS), a standardized child development screening tool designed to identify children at risk for developmental, social, or behavioral delay (Glascoe, 2003).^3^ First, we assess concerns about early learning by taking the sum of parents’ responses to whether they are a lot (2), a little (1), or not at all (0) concerned about their child’s school skills, speech, and ability to do things by themselves. Second, we assess concerns about socio-emotional development by taking the sum of parents’ responses to whether they are a lot (2), a little (1), or not at all (0) concerned about their child’s behavior and ability to get along with others. Third, we assess concerns about physical health and motor development by taking the sum of parents’ responses to whether they are a lot (2), a little (1), or not at all (0) concerned about their child’s ability to use their hands and fingers and arms and legs (Cronbach’s alpha = 0.84, 0.81, and 0.86). We standardized each index (*M* = 0, *SD* = 1).

### Independent Variables

Our independent variables of interest are measures of state health and educational spending before age six. State spending data come from the Urban Institute’s State-by-State Spending on Kids Dataset from 1998 to 2016(Isaacs et al., 2020) and public pre-K spending data come from the National Institute for Early Education Research’s (NIEER) 2004, 2008, and 2012 State Preschool Yearbooks. State spending measures include spending in states from federal (e.g., Head Start, federal Medicaid funding), state, and local sources (e.g., state- and locally sponsored prekindergarten, state Medicaid funds). Importantly, our measure of Medicaid spending specifically measures the “kids’ share” of Medicaid.

We merge these spending data with child-level data from the NSCH using state and year identifiers to examine the effects of public child-directed spending on children’s school readiness. The State-by-State Spending on Kids Dataset reports spending by calendar year, and the NIEER State Preschool Yearbooks report spending by school year. Because the NSCH does not include identifiers for the year in which a respondent was surveyed, we use the first year of the survey wave as the year identifier (see Appendix).

We use $1,000 in spending per child 0–18 in 2016 dollars. Our measure of health spending represents total public spending on Medicaid for children and CHIP, public health efforts, and health vendor payments and public hospitals (excluding Medicaid). Our measure of spending on early education represents federal spending on Head Start awarded to private grantees and state spending on public pre-K.

To control for other factors that may be correlated with parent concerns, we include several parent-reported covariates, including the child’s race/ethnicity (non-Hispanic White, non-Hispanic Black, Hispanic, non-Hispanic other), age (in years), and gender (male or female); the number of children under 18 in the household; the parent’s marital status (unmarried or married [marital status and cohabitation are reported differently across survey waves; for the 2003/2004 wave, we use the number of adults in the household as an indicator of marital status. For the 2007/2008 and 2011/2012 waves, we consider cohabitating and/or legally married parents to be married]) and parent gender (male or female); whether someone in the household was employed at least 50 out of the past 52 weeks; the highest level of education of the parents (more than a high school degree, a high school degree, or less than a high school degree); and the household’s federal poverty level (FPL) percent (more than 400%, 300–400%, 200–300%, 185–200%, 150–185%, 133–150%, 100–133%, or less than or at 100%).

Annual state-level characteristics come from the American Community Survey, the University of Kentucky Center for Poverty Research National Welfare Database (UKCPR, 2023), and the Current Population Survey, merged using state and year identifiers. These characteristics include state unemployment rate, poverty rate, the proportion of adults with a college degree or more, the proportion who were non-Hispanic Black, the proportion who were Hispanic, whether the state has a Democratic governor, and the state minimum wage.

### Analytic Strategy

To identify the association between state spending and parent-reported concerns about their children’s school readiness, we use ordinary least squares (OLS) regression to predict parents’ concerns regarding their children’s early learning, socio-emotional development, or physical health and motor development from state-level health and early education spending, controlling for household- and state-level characteristics, and state and survey wave fixed effects. We test for heterogeneity by educational attainment, income, and race by including interactions between spending and (respectively) parent education, household FPL, and child race and ethnicity. We also examine how state spending predicts Medicaid/CHIP and Head Start participation and whether these associations explain our primary findings. See the Appendix for details.

## Results

Table 1 displays sample descriptive statistics. State public spending on children’s health averages $6,890 per child per year (in 2016 dollars) for our analytic sample of children across the 50 states surveyed in 2003/2004, 2007/2008, and 2011/2012. State spending on early education is much lower, averaging $140 per child per year. There is considerable variation across and within states in the level of spending on in-kind health and early childhood educational supports, with a more than 6-fold difference between the highest and lowest state-years for health spending and a nearly 4-fold difference between the highest and lowest state-years for early education spending.

**Table 1:** Descriptive statistics of households in the analytic sample

**Household Characteristics**	
Highest level of education of parent(s)	
More than HS	59.40%
HS	23.50%
Less than HS	17.10%
Parent race/ethnicity	
NH White	55.81%
NH Black	11.92%
Hispanic	22.79%
NH Other	9.48%
Parent unmarried	14.26%
Parent is mother	82.55%
Someone in the HH was employed at least 50/52 weeks	87.54%
Number of HH children	2.29
	(0.93)
Child age (in years)	3.31
	(1.30)
Child female	49.02%
FPL	
400%+	27.12%
300–400%	12.59%
200–300%	16.35%
185–200%	3.04%
150–185%	7.14%
133–150%	3.47%
100–133%	8.64%
Less than 100%	21.64%
Public Program Participation	
Medicaid/CHIP	37.06%
Head Start/Early Start	12.39%
**Dependent Variables**	
Standardized early learning	0.04
	(1.06)
Standardized socio-emotional development	0.08
	(1.07)
Standardized physical health and motor development	0.02
	(1.03)
**State Spending**	
2003, 2007, 2011 Health spending ($1,000 per child 0–18, 2016 dollars)	6.89
	(1.94)
2003 Health spending	5.99
	(1.45)
2007 Health spending	6.65
	(1.74)
2011 Health spending	8.09
	(1.99)
2003, 2007, 2011 Early education spending ($1,000 per child 0–18, 2016 dollars)	0.14
	(0.06)
2003 Early education spending	0.12
	(0.05)
2007 Early education spending	0.14
	(0.06)
2011 Early education spending	0.14
	(0.06)
**Observations**	56,736

Source/Notes: Authors’ analysis from 2003/2004, 2007/2008, and 2011/2012 Waves of the National Survey of Children’s Health, Urban Institute’s State-by-State Spending on Kids Dataset from 1998 to 2016, and National Institute for Early Education Research’s 2004, 2008, and 2012 State Preschool Yearbook. HS is high school. NH is non-Hispanic. HH is household. FPL is federal poverty level. Standard deviations are in parentheses. Head Start/Early Start participation is only reported in the 2003/2004 wave (*N* = 21,012)

About three in five children have parents with more than a high school degree, and more than half (56%) are White whereas about one-quarter (23%) are Hispanic, 12% non-Hispanic Black, and just under 10% are another race/ethnicity. The majority of respondent parents are mothers (83%), and few (14%) are unmarried. Nearly nine in ten (88%) children live in a household where one or more adults are employed over the year. More than one-fifth (22%) of children live in households below the poverty line, whereas 27% live in households above 400% FPL. More than one-third (37.06%) of children are covered by Medicaid/CHIP, and fewer (12.39%) participate in Head Start or Early Start.

Table 2 displays the main regression analyses. In our preferred models, which include state and year fixed effects and state-level time-varying covariates, we see in Panel A that an increase of $1,000 in state child-related health spending is associated with a small reduction in parents’ concerns about children’s physical and motor development (-0.04 of a SD). Likewise, as shown in Panel B, an increase of $1,000 in state early education spending is associated with a reduction in parents’ concerns about children’s physical and motor development. The coefficient is quite large (-4.14 SD). In preferred models, neither health spending nor early education spending are significantly associated with concerns about early learning or social-emotional development.

**Table 2:** OLS regression results showing the association between spending and parents' concerns about child's development.

	(1)	(2)	(3)	(4)	(5)	(6)
	Early Learning	Early Learning	Socio-emotional Development	Socio-emotional Development	Physical Health & Motor Development	Physical Health & Motor Development
*Panel A: Health Spending*						
Health spending ($1,000 per child 0–18, 2016 dollars)	0.00711	-0.0289	0.00429	-0.0293	0.00265	-0.0440**
	(0.00502)	(0.0260)	(0.00538)	(0.0192)	(0.00671)	(0.0144)
Individual covariates	Yes	Yes	Yes	Yes	Yes	Yes
Year FE	No	Yes	No	Yes	No	Yes
State covariates	No	Yes	No	Yes	No	Yes
State FE	No	Yes	No	Yes	No	Yes
Constant	-0.0322	2.477*	0.120	2.144*	0.0763	1.854**
	(0.0689)	(1.079)	(0.0740)	(1.065)	(0.0663)	(0.673)
Observations	56,736	56,736	56,736	56,736	56,736	56,736
*Panel B: Early Education Spending*						
Early education spending($1,000 per child 0–18, 2016 dollars)	1.081*	-5.396	0.685	-3.725	1.380**	-4.142*
	(0.449)	(3.656)	(0.563)	(2.996)	(0.328)	(1.896)
Individual covariates	Yes	Yes	Yes	Yes	Yes	Yes
Year FE	No	Yes	No	Yes	No	Yes
State covariates	No	Yes	No	Yes	No	Yes
State FE	No	Yes	No	Yes	No	Yes
Constant	-0.0564	2.464**	0.103	1.961*	-0.00182	1.440**
	(0.0584)	(0.900)	(0.0765)	(0.920)	(0.0636)	(0.515)
Observations	56,736	56,736	56,736	56,736	56,736	56,736

Source/Notes: Authors’ analysis from 2003/2004, 2007/2008, and 2011/2012 Waves of the National Survey of Children’s Health, Urban Institute’s State-by-State Spending on Kids Dataset from 1998 to 2016, and National Institute for Early Education Research’s 2004, 2008, and 2012 State Preschool Yearbook. ** *p* < 0.01, * *p* < 0.05, + *p* < 0.1

**Fig. 1:**
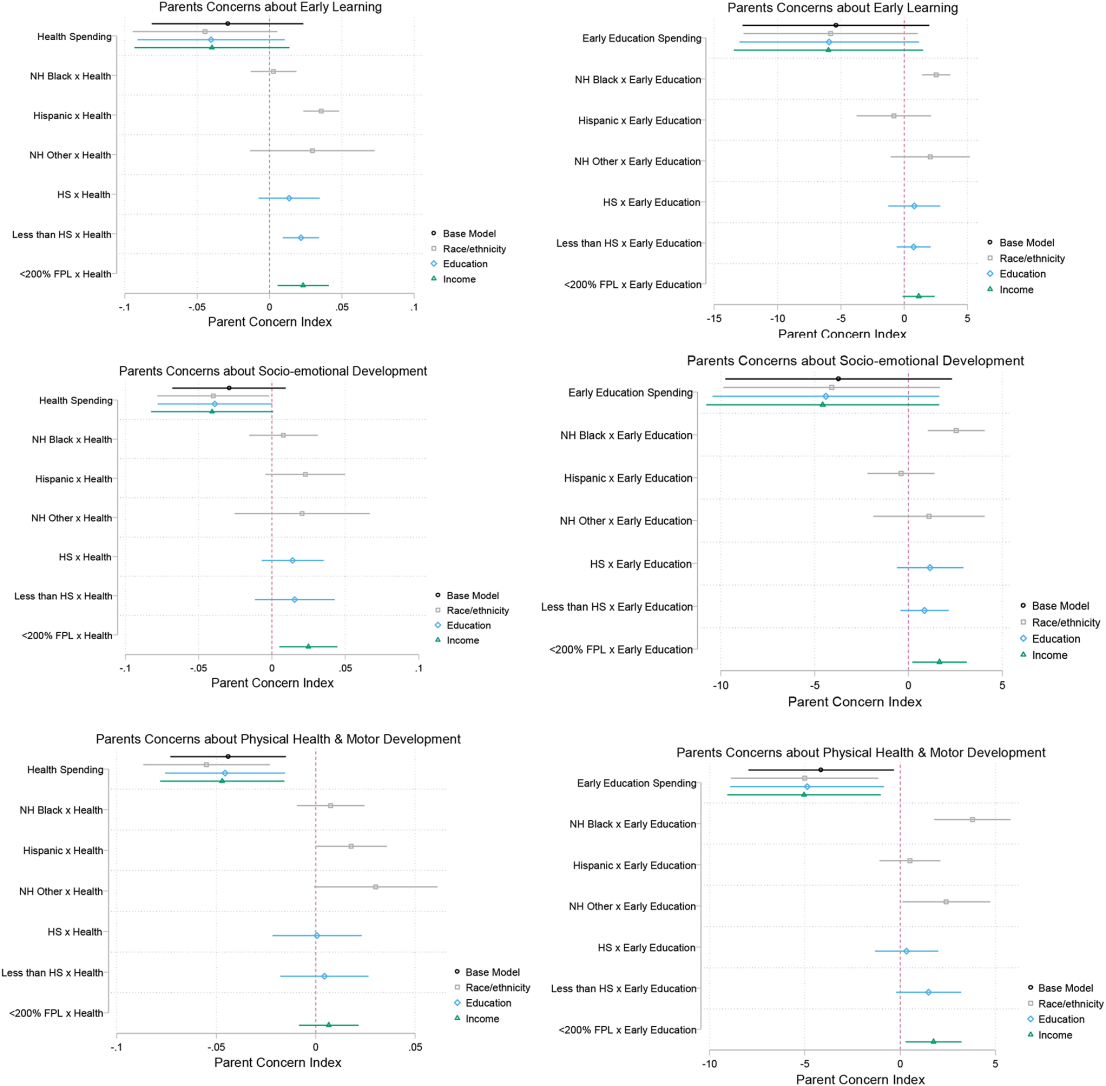
Coefficients on the health/education spending variable in OLS regressions. Source/Notes: Authors’ analysis from 2003/2004, 2007/2008, and 2011/2012 Waves of the National Survey of Children’s Health, Urban Institute’s State-by-State Spending on Kids Dataset from 1998 to 2016, and National Institute for Early Education Research’s 2004, 2008, and 2012 State Preschool Yearbook. Each panel shows regression coefficients for models regressing indicated outcome variable on spending (base model) and for models regressing indicated outcome variable on interaction of spending and each household characteristic. All models include individual and state covariates and year and state fixed effects. HS is high school. NH is non-Hispanic. HH is household. FPL is federal poverty level

Figure 1 shows coefficient plots for the main associations between health and early learning spending and parent-reported concerns, and the interactive effects for subgroups to test for heterogeneity by parental educational attainment, income, and race. Appendix Tables A1, A2, and A3 show the full regression model results. While the overall associations between public spending (health or early education) and parents’ concerns are negative and largely insignificant, among disadvantaged and racial/ethnic minority groups, more spending is associated with smaller reductions in parents’ concerns. For example, parents of Hispanic children, those lacking a high school degree, and those under 200% FPL show significantly different and smaller associations between health spending and concerns about children’s early learning than their white, more educated, or higher-income counterparts. Associations between early education spending and parents’ concerns are more heterogeneous than associations with health spending. Among low-income parents and parents of non-Hispanic Black children, greater early education spending is associated with smaller reductions in concerns about children’s social-emotional development and health, and this is also true for early learning concerns among parents of non-Hispanic Black children. Notably, state-level health spending on children is consistently related to reductions in parents’ concerns about children’s physical health and motor development across all subgroups. Public spending on early education is positively associated with participation rates in Medicaid/CHIP and in Head Start/Early Head Start, respectively, although the health spending coefficient is not significant (see the Appendix and Appendix Table A4 for details).

## Discussion

This study is among the first to examine how state-level public investments in in-kind supports for children are associated with measures of young children’s health and well-being. We use a large, nationally- and state-representative dataset, combining multiple sources of publicly available data to connect policy decisions to family characteristics and children’s development. Specifically, we examine parents’ reports of children’s early learning, social-emotional development, and physical health and motor development, all of which are important aspects of children’s readiness for kindergarten and strong predictors of health and well-being later in life (Duncan et al., 2007). Importantly, these measures go beyond physical health status to encompass a range of crucial developmental areas, including cognitive and social development. Across all families, we find that higher levels of public health and early education spending are associated with decreases in concerns about children’s physical health and motor development, but largely unassociated with concerns about other areas of development. However, reductions in concerns are smaller among groups of parents historically considered disadvantaged– those with low incomes, low levels of education, or who are racial or ethnic minorities– relative to their more advantaged counterparts.

The association between public spending and reductions in parents’ health concerns was much larger for early education spending than health spending, which may stem from differing relative levels of public investments in these areas as well as the historical period. In our study period (2003–2012), health spending averaged $6,890 per child per year (in 2016 dollars) with a standard deviation of nearly $2,000. Public spending in early education was much lower and showed less variation over this period, averaging just $140 per child per year. We find that a $1,000 increase in health spending per child per year, or about a 15% increase in average spending, is associated with a reduction in parents’ concerns about children’s health and motor development of about 3% of a SD. The coefficient between early education spending and health concerns was much larger, with $1,000 per child per year associated with a decrease in concerns of more than 4.1 SDs; notably, though, a $1,000 increase represents a 714% increase over average spending. A comparable 15% increase in early education spending would be $21 per child per year, and assuming the association between early education spending and parents’ concerns was linear, this would translate into a 2% of a SD reduction in parents’ concerns. This finding also emphasizes the importance of public spending and programming beyond traditional health or medical care for health and other outcomes.

There is considerable variation across time and states in public spending on children’s health. Head Start enrollment and spending have remained relatively steady since the early 2000s at just under one million children, but state-level prekindergarten investments have increased dramatically. In 2003, state-funded preschool programs enrolled about 16% of all four year-olds in the U.S., compared to 28% in 2012 (Barnett et al., 2012). Medicaid/CHIP enrollment also grew; in 2003, approximately half of all low-income children (under 200% FPL) were enrolled in Medicaid or CHIP, increasing to 63% in 2012 (Mathematica Policy Research & The Urban Institute, 2014). Similarly, although both health spending and early education spending increased in 2003, 2007, and 2011, health spending increased by about 35% from 2003 to 2011, whereas early education spending increased by only about 17% that same period. Associations between state public spending and parents’ concerns about their child’s development may vary during different economic or political contexts.

Another key finding is that the associations between early education spending and parents’ health concerns are smaller among racial and ethnic minority parents. An increase of $1,000 per child in early education spending is associated with a decrease in concerns of 1.6 SDs among parents of non-Hispanic Black children and 2.6 SDs among parents of non-Hispanic other race children, compared to a 5.0 SD decrease among parents of non-Hispanic White children. Associations between more health spending and reductions in health concerns were consistent among parents across races, ethnicities, and measures of SES.

Despite the importance of public early educational investments for children’s health and development, their reach is more limited than that of public health insurance; although early education spending is positively associated with Head Start/Early Start enrollment (in 2003 when data are available), only 13% of the sample reports that their children attend. It is possible that higher levels of spending increase slots or opportunity, but not enough to overcome the limited resources and meaningfully narrow large racial, ethnic, and SES gaps in early education attendance or school readiness (Magnuson & Waldfogel, 2016; Reardon & Portilla, 2016). It is also possible that greater participation in public health insurance and early education programs provide parents with comparisons to their own children and serve a role in educating participants about developmental benchmarks for their children. Relatedly, public health and early education programs screen young children for problems, identifying and treating them earlier; thus, what may appear as a parent concern about a young child may indicate early identification, beneficial for long-term outcomes. While participation in early childhood education programs broadly (public and private) has become the norm, private programs are expensive and in short supply, especially for families with lower incomes and for infants and toddlers (Chaudry et al., 2021).

Our study has several limitations. First, our findings are not causal; parent concerns might affect decisions about whether to live in higher (or lower) spending states. Parents who choose to reside in higher-spending states may do so because they are seeking a certain level of investment in their children’s physical health or motor development, a reverse relationship from the one we attempt to isolate. Relatedly, an important area for investigation remains how parents’ concerns about their children’s development affects public spending on children, via voting behaviors or other types of activism. Second, our measures of school readiness stem from parents’ reported concerns and may therefore not reflect differences in children’s actual skills, diagnoses, or abilities (though the measures we use are well-validated (Glascoe, 2003)); newer waves of the NSCH (2016 and after) contain parent-reported measures of specific skills (Ghandour et al., 2019) and should be examined in future research. The present study is unable to do so, as the state spending data available publicly end in 2016. Notably, however, the importance of family engagement in developmental monitoring is emphasized by both the American Academy of Pediatrics and the Centers for Disease Control and Prevention (Abercrombie et al., 2022; Lipkin & Macias, 2020). An important area for future research, while resource-intensive, is gathering post-2016 data to examine state spending during and following the COVID-19 pandemic. Given the dramatic but temporary increase in child-related public spending, studying the period surrounding and following the pandemic may be particularly helpful in understanding the macro-level drivers of children’s health and development. The data used here represent the most recent state-level data on social safety net investments in children that are publicly available.

In sum, our study adds to growing evidence that state-level variation in public spending on children– specifically for health and early education– may have meaningful implications for children’s early health and development (Jackson et al., 2022). More research is needed to understand the mechanisms underlying these associations, and how these associations may vary across social and historical contexts.

## Electronic Supplementary Material

Below is the link to the electronic supplementary material.

Supplementary Material 1

## Data Availability

The National Survey of Children’s Health (NSCH) data are made publicly available by the Maternal and Child Health Bureau at the Health Resources and Services Administration (HRSA). See: https://www.childhealthdata.org/learn-about-the-nsch/NSCH.

## Abbreviations

SES: Socioeconomic status
NSCH: National Survey of Children’s Health
NIEER: National Institute of Early Education Research

## Footnotes

1 Prior to a survey redesign in 2016, the NSCH was conducted by the U.S. Department of Health and Human Services, Maternal and Child Health Bureau of Health Resources and Services Administration and the Office of the Assistant Secretary for Planning and Evaluation of the Department of Health and Human Services.

2 Because data on public pre-K spending is not available for Washington, DC, in 2003–2004 and 2007–2008, we do not include respondents from DC in our analytic sample.

3 See also: https://www.childhealthdata.org/docs/nsch-docs/sas-codebook_-2011-2012-nsch-v1_05-10-13.pdf.

## Code Availability

The authors will make Stata code available by request.
