# Supplementary material for: State-Level Spending on Children Associated with Unequal Benefits to School Readiness
Source: Matern Child Health J. 2025 Feb 14;29(4):494–503. doi: 10.1007/s10995-025-04068-9 (PMC12006267; doi:10.1007/s10995-025-04068-9)
Supplement: Supplementary file 1 — Supplementary Material 1 [file 10995_2025_4068_MOESM1_ESM.docx]

**Appendix**

***Years of Data Matched***

For the Urban Institute’s State-by-State Spending on Kids data, 2003 spending data are merged with 2003/2004 survey data, 2007 spending data are merged with 2007/2008 survey data, and 2011 spending data are merged with 2011/2012 survey data. In sensitivity analyses, we test the robustness of our findings using spending in different years. NIEER does not have a yearbook dedicated to the 2003-2004 school year, so we merge spending data from the 2002-2003 school year, the 2007-2008 school year, and the 2011-2012 school year with the 2003/2004, 2007/2008, and 2011/2012 waves of the NSCH, respectively.

***Imputation in the National Survey of Children’s Health***

The NSCH imputes income and other household characteristics when respondents do not provide valid family size, total family income, or other critical information, creating five implicates that can be used to estimate measures like household poverty in cases in which the inputs are not directly observed. Missing data are non-trivial; in 2003, prior to imputation, 9.2% of the total survey observations were missing at least one variable needed to calculate respondents’ federal poverty level (FPL) percent (1).

***Analytic Strategy***

To identify the association between state spending and parent-reported concerns about their children’s school readiness, we estimate the following model using ordinary least squares (OLS) regression:

1. $Y_{chsy}=\alpha+{\beta_{1}Spend}_{sy}+\beta_{2}X_{h}+\mu_{s}+ \rho_{sy}+\phi_{y}+\epsilon_{chsy}$

Here, $Y_{chsy}$ is a measure of school readiness based on parent concern about child c in household h in state s in year y – either concern about early learning, socio-emotional development, or physical health and motor development. ${Spend}_{sy}$ is a measure of spending on either health or early education in state s in year y. $X_{h}$ is a vector of household-level covariates. In our preferred specification, we also include state fixed effects, $\mu_{s}$, to control for time-invariant differences across geography, and a vector of state covariates,$\rho_{sy}$, to control for time-varying state characteristics. Because the NSCH does not include identifiers for the specific year in which a respondent was surveyed (only the survey wave, which occurs over two years), we include survey wave fixed effects, $\phi_{y}$, to control for secular time trends (hereafter called “year fixed effects”).

***Examining State Spending and Public Program Participation***

Because a primary pathway by which public spending may affect children’s outcomes is through increased participation in public benefits, we examine whether state spending was associated with parents’ reports of whether the child was covered by Medicaid or CHIP (available in all waves) and the child’s Head Start or Early Start program attendance (collected in the 2003/2004 survey wave only, N=21,012). Finally, we measure Medicaid/CHIP participation in our health spending models, and Head Start/Early Start participation in early education spending models (2003/2004 wave only), to examine whether program participation explains any of the (expected) associations between state spending and parents’ concerns about their children’s school readiness.

Results from these analyses indicate that, as expected, public spending on health and early education is positively associated with participation rates in Medicaid/CHIP and in Head Start/Early Head Start, respectively, although the health spending coefficient is not significant (see Appendix Exhibit A4). A $1,000 increase in early education spending (a very large increase relative to the mean of $140) is associated with a 71.2 percentage point increase in the likelihood of attending Head Start/Early Start. Likewise, pairwise correlations indicate that health spending and Medicaid/CHIP participation (*r=*0.46), and early education spending and Head Start/Early Start participation (*r*=0.64), are highly correlated. These findings increase our confidence that these state spending measures are proxies for families’ access to and participation in public benefits, particularly early education.

Next, we add participation in public programs as covariates to our main models predicting parent-reported concerns. Results indicate that children’s participation in Medicaid/CHIP is associated with more parent-reported concerns about children’s early learning and social-emotional development, and health spending is no longer related to parents’ concerns (see Appendix Exhibit A5: Panel A). Health spending predicts reductions in parents’ concerns about children’s physical health and motor development, with Medicaid/CHIP participation marginally significantly associated with more parental concerns (potentially a selection effect regarding public health insurance enrollment). In contrast, both state early education spending and Head Start/Early Start participation predict higher levels of parent concerns across all domains (Appendix Exhibit A5: Panel B). Importantly, though, the early learning models are estimated from a subset of the broader sample (2003-2004 only).

***Sensitivity Analyses***

Finally, we conduct sensitivity analyses examining different years of state spending. Because we only observe the survey wave, not the exact year of survey, in the main analysis (Exhibit 2), we use spending data for the first year of the NSCH panel wave (2003, 2007, and 2011). In Appendix Exhibit A6 we show models that measure state spending in the second year of the wave (2004, 2008, and 2012). The results of this analysis are consistent with our main findings, ameliorating concerns about our inability to observe the precise time at which a parent is surveyed. Further, models displayed in Appendix Exhibit A7 use state spending in the year prior to the first year of the wave (2002, 2006, and 2010) to identify whether parents’ concerns are predicted by earlier years of spending. Unlike our main findings, the results with full controls show no statistically significant association between health or education spending and concerns about children’s physical health and motor development, although the coefficients are negative. This suggests that spending may have an immediate or short-term effect on children and/or declines in concerns associated with increases in spending may not persist over time.

**Table A1.** OLS regression results showing the association between spending and concerns about child development, by parent educational attainment.

|  | (1) | (2) | (3) | (4) | (5) | (6) |
| --- | --- | --- | --- | --- | --- | --- |
|  | Early Learning | Early Learning | Socio-emotional Development | Socio-emotional Development | Physical Health & Motor Development | Physical Health & Motor Development |
| *Panel A: Health Spending* |  |  |  |  |  |  |
| Health spending ($1,000 per child 0-18, 2016 dollars) | 0.000736 | -0.0403 | -0.000838 | -0.0389+ | 0.00369 | -0.0455** |
|  | (0.00361) | (0.0252) | (0.00479) | (0.0194) | (0.00491) | (0.0150) |
| More than HS | Omitted | Omitted | Omitted | Omitted | Omitted | Omitted |
| HS | -0.0310 | -0.0307 | -0.0570 | -0.0538 | 0.0869 | 0.0822 |
|  | (0.0993) | (0.0948) | (0.0857) | (0.0852) | (0.0997) | (0.0973) |
| Less than HS | 0.0329 | 0.0135 | 0.0660 | 0.0274 | 0.126 | 0.108 |
|  | (0.0561) | (0.0519) | (0.0733) | (0.0997) | (0.0952) | (0.0816) |
| HS*Health spending | 0.0122 | 0.0138 | 0.0133 | 0.0140 | -0.00230 | 0.000704 |
|  | (0.0110) | (0.0105) | (0.0108) | (0.0105) | (0.0112) | (0.0111) |
| Less than HS*Health spending | 0.0179** | 0.0218** | 0.00930 | 0.0155 | -0.00249 | 0.00439 |
|  | (0.00622) | (0.00627) | (0.0104) | (0.0135) | (0.0129) | (0.0110) |
| Individual covariates | Yes | Yes | Yes | Yes | Yes | Yes |
| Year FE | No | Yes | No | Yes | No | Yes |
| State covariates | No | Yes | No | Yes | No | Yes |
| State FE | No | Yes | No | Yes | No | Yes |
| Constant | 0.00897 | 2.486* | 0.154* | 2.161+ | 0.0696 | 1.850** |
|  | (0.0598) | (1.105) | (0.0697) | (1.084) | (0.0582) | (0.682) |
| Observations | 56,736 | 56,736 | 56,736 | 56,736 | 56,736 | 56,736 |
|  |  |  |  |  |  |  |
| *Panel B: Early Education Spending* |  |  |  |  |  |  |
| Early education spending ($1,000 per child 0-18, 2016 dollars) | 0.800* | -5.930+ | 0.256 | -4.408 | 1.102** | -4.875* |
|  | (0.363) | (3.516) | (0.491) | (2.998) | (0.322) | (2.000) |
| More than HS | Omitted | Omitted | Omitted | Omitted | Omitted | Omitted |
| HS | -0.00295 | 0.00773 | -0.0511 | -0.0399 | 0.0472 | 0.0623 |
|  | (0.0983) | (0.0953) | (0.0810) | (0.0807) | (0.0808) | (0.0787) |
| Less than HS | 0.131** | 0.115** | 0.0814* | 0.0758 | 0.0257 | 0.0328 |
|  | (0.0588) | (0.0524) | (0.0482) | (0.0551) | (0.0603) | (0.0629) |
| HS* Early education spending | 0.797 | 0.781 | 1.209 | 1.149 | 0.316 | 0.340 |
|  | (1.043) | (1.016) | (0.891) | (0.879) | (0.832) | (0.822) |
| Less than HS*Early education spending | 0.488 | 0.744 | 0.752 | 0.856 | 1.192 | 1.488+ |
|  | (0.677) | (0.664) | (0.585) | (0.633) | (0.799) | (0.847) |
| Individual covariates | Yes | Yes | Yes | Yes | Yes | Yes |
| Year FE | No | Yes | No | Yes | No | Yes |
| State covariates | No | Yes | No | Yes | No | Yes |
| State FE | No | Yes | No | Yes | No | Yes |
| Constant | -0.0367 | 2.471** | 0.133+ | 1.969* | 0.0171 | 1.455* |
|  | (0.0548) | (0.916) | (0.0680) | (0.941) | (0.0644) | (0.544) |
| Observations | 56,736 | 56,736 | 56,736 | 56,736 | 56,736 | 56,736 |

Source/Notes: Authors’ analysis from 2003/2004, 2007/2008, and 2011/2012 Waves of the National Survey of Children’s Health, Urban Institute’s State-by-State Spending on Kids Dataset from 1998 to 2016, and National Institute for Early Education Research’s 2004, 2008, and 2012 State Preschool Yearbook. HS indicates high school diploma. ** p<0.01, * p<0.05, + p<0.1

**Table A2.** OLS regression results showing the association between spending and concerns about child development, by family income.

|  | (1) | (2) | (3) | (4) | (5) | (6) |
| --- | --- | --- | --- | --- | --- | --- |
|  | Early Learning | Early Learning | Socio-emotional Development | Socio-emotional Development | Physical Health & Motor Development | Physical Health & Motor Development |
| *Panel A: Health Spending* | -0.00354 | -0.0397 | -0.00637 | -0.0408+ | -0.000631 | -0.0470** |
| Health spending ($1,000 per child 0-18, 2016 dollars) | (0.00717) | (0.0266) | (0.00817) | (0.0207) | (0.00767) | (0.0155) |
|  | 0.0843 | 0.102+ | 0.0768 | 0.0798 | 0.0975 | 0.105+ |
| >=200% FPL | Omitted | Omitted | Omitted | Omitted | Omitted | Omitted |
| <200% FPL | (0.0542) | (0.0570) | (0.0677) | (0.0712) | (0.0596) | (0.0609) |
|  | 0.0241** | 0.0234** | 0.0241** | 0.0248* | 0.00742 | 0.00657 |
| <200% FPL*Health spending | (0.00838) | (0.00862) | (0.00896) | (0.00977) | (0.00704) | (0.00731) |
|  |  |  |  |  |  |  |
| Individual covariates | Yes | Yes | Yes | Yes | Yes | Yes |
| Year FE | No | Yes | No | Yes | No | Yes |
| State covariates | No | Yes | No | Yes | No | Yes |
| State FE | No | Yes | No | Yes | No | Yes |
| Constant | 0.0385 | 2.524* | 0.191* | 2.193* | 0.0981 | 1.867** |
|  | (0.0705) | (1.096) | (0.0874) | (1.082) | (0.0733) | (0.676) |
| Observations | 56,736 | 56,736 | 56,736 | 56,736 | 56,736 | 56,736 |
|  |  |  |  |  |  |  |
| *Panel B: Early Education Spending* |  |  |  |  |  |  |
| Early education spending ($1,000 per child 0-18, 2016 dollars) | 0.466 | -5.982 | -0.162 | -4.570 | 0.521 | -5.039* |
|  | (0.477) | (3.708) | (0.665) | (3.084) | (0.513) | (1.997) |
| >=200% FPL | Omitted | Omitted | Omitted | Omitted | Omitted | Omitted |
| <200% FPL | 0.154* | 0.182* | 0.116+ | 0.134+ | 0.0147 | 0.0251 |
|  | (0.0684) | (0.0763) | (0.0677) | (0.0698) | (0.0655) | (0.0683) |
| <200% FPL*Early education spending | 1.250* | 1.142+ | 1.721* | 1.648* | 1.744* | 1.748* |
|  | (0.594) | (0.619) | (0.695) | (0.712) | (0.720) | (0.724) |
| Individual covariates | Yes | Yes | Yes | Yes | Yes | Yes |
| Year FE | No | Yes | No | Yes | No | Yes |
| State covariates | No | Yes | No | Yes | No | Yes |
| State FE | No | Yes | No | Yes | No | Yes |
| Constant | -0.0136 | 2.496** | 0.162+ | 2.007* | 0.0579 | 1.489** |
|  | (0.0642) | (0.906) | (0.0823) | (0.933) | (0.0742) | (0.527) |
| Observations | 56,736 | 56,736 | 56,736 | 56,736 | 56,736 | 56,736 |

Source/Notes: Authors’ analysis from 2003/2004, 2007/2008, and 2011/2012 Waves of the National Survey of Children’s Health, Urban Institute’s State-by-State Spending on Kids Dataset from 1998 to 2016, and National Institute for Early Education Research’s 2004, 2008, and 2012 State Preschool Yearbook. FPL is Federal Poverty Level. ** p<0.01, * p<0.05, + p<0.1

**Table A3.** OLS regression results showing the association between spending and concerns about child development, by race/ethnicity.

|  | (1) | (2) | (3) | (4) | (5) | (6) |
| --- | --- | --- | --- | --- | --- | --- |
|  | Early Learning | Early Learning | Socio-emotional Development | Socio-emotional Development | Physical Health & Motor Development | Physical Health & Motor Development |
| *Panel A: Health Spending* |  |  |  |  |  |  |
| Health spending ($1,000 per child 0-18, 2016 dollars) | -0.00367 | -0.0444+ | -0.00170 | -0.0400* | -0.00400 | -0.0549** |
|  | (0.00349) | (0.0248) | (0.00557) | (0.0190) | (0.00547) | (0.0158) |
| NH White | Omitted | Omitted | Omitted | Omitted | Omitted | Omitted |
| NH Black | 0.201* | 0.220** | 0.111 | 0.129 | 0.139 | 0.153* |
|  | (0.0783) | (0.0661) | (0.107) | (0.0943) | (0.0866) | (0.0721) |
| Hispanic | -0.0407 | -0.101* | -0.0289 | -0.121 | 0.0692 | 0.00676 |
|  | (0.0522) | (0.0499) | (0.0567) | (0.0839) | (0.0731) | (0.0546) |
| NH Other | 0.0131 | -0.0236 | 0.0667 | 0.0316 | -0.0705 | -0.112 |
|  | (0.149) | (0.145) | (0.165) | (0.153) | (0.116) | (0.108) |
| NH Black*Health spending | 0.00906 | 0.00287 | 0.0125 | 0.00776 | 0.0134 | 0.00754 |
|  | (0.0101) | (0.00786) | (0.0137) | (0.0116) | (0.0105) | (0.00842) |
| Hispanic*Health spending | 0.0307** | 0.0359** | 0.0120 | 0.0227 | 0.0108 | 0.0179+ |
|  | (0.00629) | (0.00614) | (0.00919) | (0.0136) | (0.0119) | (0.00891) |
| NH Other*Health spending | 0.0264 | 0.0298 | 0.0167 | 0.0205 | 0.0246 | 0.0302+ |
|  | (0.0221) | (0.0214) | (0.0244) | (0.0229) | (0.0165) | (0.0154) |
| Individual covariates | Yes | Yes | Yes | Yes | Yes | Yes |
| Year FE | No | Yes | No | Yes | No | Yes |
| State covariates | No | Yes | No | Yes | No | Yes |
| State FE | No | Yes | No | Yes | No | Yes |
| Constant | 0.0374 | 2.442* | 0.160* | 2.128+ | 0.122+ | 1.843** |
|  | (0.0658) | (1.073) | (0.0755) | (1.074) | (0.0699) | (0.680) |
| Observations | 56,736 | 56,736 | 56,736 | 56,736 | 56,736 | 56,736 |
|  |  |  |  |  |  |  |
| *Panel B: Early Education Spending* |  |  |  |  |  |  |
| Early education spending($1,000 per child 0-18, 2016 dollars) | 0.442+ | -5.817+ | 0.0388 | -4.096 | 0.274 | -5.009* |
|  | (0.250) | (3.413) | (0.408) | (2.863) | (0.305) | (1.918) |
| NH White | Omitted | Omitted | Omitted | Omitted | Omitted | Omitted |
| NH Black | 0.0422 | 0.0450 | -0.0279 | -0.0119 | -0.0615 | -0.0829 |
|  | (0.0477) | (0.0432) | (0.0720) | (0.0703) | (0.0790) | (0.0751) |
| Hispanic | 0.242** | 0.193** | 0.100 | 0.0557 | 0.112** | 0.0889 |
|  | (0.0944) | (0.0944) | (0.0676) | (0.0620) | (0.0537) | (0.0551) |
| NH Other | 0.0684 | 0.0374 | 0.115 | 0.0962 | -0.0589 | -0.0740 |
|  | (0.106) | (0.100) | (0.112) | (0.105) | (0.0806) | (0.0746) |
| NH Black*Early education spending | 2.827** | 2.518** | 2.923** | 2.545** | 3.782** | 3.785** |
|  | (0.585) | (0.552) | (0.772) | (0.752) | (1.020) | (0.996) |
| Hispanic*Early education spending | -1.119 | -0.826 | -0.731 | -0.396 | 0.517 | 0.507 |
|  | (1.543) | (1.455) | (1.009) | (0.887) | (0.873) | (0.794) |
| NH Other*Early education spending | 1.839 | 2.045 | 0.984 | 1.086 | 2.285+ | 2.414* |
|  | (1.578) | (1.544) | (1.530) | (1.474) | (1.189) | (1.140) |
| Individual covariates | Yes | Yes | Yes | Yes | Yes | Yes |
| Year FE | No | Yes | No | Yes | No | Yes |
| State covariates | No | Yes | No | Yes | No | Yes |
| State FE | No | Yes | No | Yes | No | Yes |
| Constant | -0.00913 | 2.457** | 0.151* | 1.952* | 0.0779 | 1.438** |
|  | (0.0512) | (0.891) | (0.0710) | (0.914) | (0.0643) | (0.524) |
| Observations | 56,736 | 56,736 | 56,736 | 56,736 | 56,736 | 56,736 |

Source/Notes: Authors’ analysis from 2003/2004, 2007/2008, and 2011/2012 Waves of the National Survey of Children’s Health, Urban Institute’s State-by-State Spending on Kids Dataset from 1998 to 2016, and National Institute for Early Education Research’s 2004, 2008, and 2012 State Preschool Yearbook. NH is non-Hispanic. ** p<0.01, * p<0.05, + p<0.1

**Table A4.** OLS regression results showing the association between spending and Medicaid/CHIP and Head Start/Early Start participation.

|  | (1) | (2) | (3) | (4) |
| --- | --- | --- | --- | --- |
|  | Medicaid Participation | Medicaid Participation | Head Start/Early Start | Head Start/Early Start |
| *Panel A: Health Spending* |  |  |  |  |
| Health spending ($1,000 per child 0-18, 2016 dollars) | 0.00357 | 0.00227 |  |  |
|  | (0.00251) | (0.0114) |  |  |
| Individual covariates | Yes | Yes |  |  |
| Year FE | No | Yes |  |  |
| State covariates | No | Yes |  |  |
| State FE | No | Yes |  |  |
| Constant | 0.0612* | 0.0870 |  |  |
|  | (0.0277) | (0.325) |  |  |
| Observations | 52,962 | 52,962 |  |  |
|  |  |  |  |  |
| *Panel B: Early Education Spending* |  |  |  |  |
| Early education spending($1,000 per child 0-18, 2016 dollars) |  |  | 0.617** | 0.712** |
|  |  |  | (0.147) | (0.154) |
| Individual covariates |  |  | Yes | Yes |
| State covariates |  |  | No | Yes |
| Constant |  |  | -0.0684** | -0.0517 |
|  |  |  | (0.0219) | (0.0477) |
| Observations |  |  | 21,012 | 21,012 |

Source/Notes: Authors’ analysis from 2003/2004, 2007/2008, and 2011/2012 Waves of the National Survey of Children’s Health, Urban Institute’s State-by-State Spending on Kids Dataset from 1998 to 2016, and National Institute for Early Education Research’s 2004, 2008, and 2012 State Preschool Yearbook. Head Start/Early Start participation data are only available for the 2003/2004 Wave of the National Survey of Children’s Health. ** p<0.01, * p<0.05, + p<0.1

**Table A5.** OLS regression results showing the association between spending and concerns about child development, controlling for public program participation.

|  | (1) | (2) | (3) | (4) | (5) | (6) |
| --- | --- | --- | --- | --- | --- | --- |
|  | Early Learning | Early Learning | Socio-emotional Development | Socio-emotional Development | Physical Health & Motor Development | Physical Health & Motor Development |
| *Panel A: Health Spending* |  |  |  |  |  |  |
| Health spending ($1,000 per child 0-18, 2016 dollars) | 0.000582 | -0.0273 | -0.00124 | -0.0221 | -0.00193 | -0.0469** |
|  | (0.00579) | (0.0267) | (0.00634) | (0.0189) | (0.00848) | (0.0172) |
| Medicaid/CHIP participant | 0.0819** | 0.0773** | 0.101** | 0.0989** | 0.0576* | 0.0522* |
|  | (0.0205) | (0.0205) | (0.0298) | (0.0311) | (0.0252) | (0.0259) |
| Individual covariates | Yes | Yes | Yes | Yes | Yes | Yes |
| Year FE | No | Yes | No | Yes | No | Yes |
| State covariates | No | Yes | No | Yes | No | Yes |
| State FE | No | Yes | No | Yes | No | Yes |
| Constant | 0.00288 | 2.264* | 0.142+ | 1.877+ | 0.0826 | 1.476* |
|  | (0.0587) | (1.067) | (0.0706) | (1.023) | (0.0662) | (0.725) |
| Observations | 52,962 | 52,962 | 52,962 | 52,962 | 52,962 | 52,962 |
|  |  |  |  |  |  |  |
| *Panel B: Early Education Spending* |  |  |  |  |  |  |
| Early education spending($1,000 per child 0-18, 2016 dollars) | 2.067** | 2.096** | 1.374** | 1.073+ | 2.037** | 1.953** |
|  | (0.346) | (0.470) | (0.334) | (0.534) | (0.438) | (0.596) |
| Head Start/Early Start participant | 0.224** | 0.223** | 0.173** | 0.173** | 0.217** | 0.216** |
|  | (0.0483) | (0.0490) | (0.0475) | (0.0472) | (0.0379) | (0.0382) |
| Individual covariates | Yes | Yes | Yes | Yes | Yes | Yes |
| State covariates | No | Yes | No | Yes | No | Yes |
| Constant | -0.111 | -0.0547 | 0.0138 | -0.0733 | -0.0604 | -0.131 |
|  | (0.0888) | (0.153) | (0.0732) | (0.144) | (0.0952) | (0.199) |
| Observations | 21,012 | 21,012 | 21,012 | 21,012 | 21,012 | 21,012 |

Source/Notes: Authors’ analysis from 2003/2004, 2007/2008, and 2011/2012 Waves of the National Survey of Children’s Health, Urban Institute’s State-by-State Spending on Kids Dataset from 1998 to 2016, and National Institute for Early Education Research’s 2004, 2008, and 2012 State Preschool Yearbook. Head Start/Early Start participation data are only available for the 2003/2004 Wave of the National Survey of Children’s Health. ** p<0.01, * p<0.05, + p<0.1

**Table A6.** OLS regression results showing the association between spending and concerns about child development, sensitivity to subsequent year of spending data.

|  | (1) | (2) | (3) | (4) | (5) | (6) |
| --- | --- | --- | --- | --- | --- | --- |
|  | Early Learning | Early Learning | Socio-emotional Development | Socio-emotional Development | Physical Health & Motor Development | Physical Health & Motor Development |
| *Panel A: Health Spending* |  |  |  |  |  |  |
| Health spending ($1,000 per child 0-18, 2016 dollars) | 0.00753 | -0.0602* | 0.00533 | -0.0361 | 0.00539 | -0.0517** |
|  | (0.00526) | (0.0244) | (0.00540) | (0.0216) | (0.00616) | (0.0133) |
| Individual covariates | Yes | Yes | Yes | Yes | Yes | Yes |
| Year FE | No | Yes | No | Yes | No | Yes |
| State covariates | No | Yes | No | Yes | No | Yes |
| State FE | No | Yes | No | Yes | No | Yes |
| Constant | -0.0366 | 2.955*** | 0.112 | 2.208** | 0.0552 | 1.911*** |
|  | (0.0704) | (1.081) | (0.0764) | (1.027) | (0.0700) | (0.644) |
| Observations | 56,736 | 56,736 | 56,736 | 56,736 | 56,736 | 56,736 |
|  |  |  |  |  |  |  |
| *Panel B: Early Education Spending* |  |  |  |  |  |  |
| Early education spending($1,000 per child 0-18, 2016 dollars) | 0.974* | -4.002 | 0.604 | -1.676 | 1.183** | -4.007+ |
|  | (0.448) | (4.292) | (0.553) | (2.812) | (0.345) | (2.269) |
| Individual covariates | Yes | Yes | Yes | Yes | Yes | Yes |
| Year FE | No | Yes | No | Yes | No | Yes |
| State covariates | No | Yes | No | Yes | No | Yes |
| State FE | No | Yes | No | Yes | No | Yes |
| Constant | -0.0489 | 2.391*** | 0.109 | 1.789** | 0.0120 | 1.490*** |
|  | (0.0602) | (0.872) | (0.0762) | (0.880) | (0.0638) | (0.500) |
| Observations | 56,736 | 56,736 | 56,736 | 56,736 | 56,736 | 56,736 |

Source/Notes: Authors’ analysis from 2003/2004, 2007/2008, and 2011/2012 Waves of the National Survey of Children’s Health, Urban Institute’s State-by-State Spending on Kids Dataset from 1998 to 2016, and National Institute for Early Education Research’s 2004, 2008, and 2012 State Preschool Yearbook. ** p<0.01, * p<0.05, + p<0.1

**Table A7.** OLS regression results showing the association between spending and concerns about child development, sensitivity to previous year of spending data.

|  | (1) | (2) | (3) | (4) | (5) | (6) |
| --- | --- | --- | --- | --- | --- | --- |
|  | Early Learning | Early Learning | Socio-emotional Development | Socio-emotional Development | Physical Health & Motor Development | Physical Health & Motor Development |
| *Panel A: Health Spending* |  |  |  |  |  |  |
| Health spending ($1,000 per child 0-18, 2016 dollars) | 0.00795 | -0.00937 | 0.00594 | -0.0113 | 0.00420 | -0.0138 |
|  | (0.00479) | (0.0209) | (0.00490) | (0.0196) | (0.00562) | (0.0140) |
| Individual covariates | Yes | Yes | Yes | Yes | Yes | Yes |
| Year FE | No | Yes | No | Yes | No | Yes |
| State covariates | No | Yes | No | Yes | No | Yes |
| State FE | No | Yes | No | Yes | No | Yes |
| Constant | -0.0356 | 2.065* | 0.110 | 1.749* | 0.0664 | 1.221* |
|  | (0.0666) | (0.795) | (0.0730) | (0.863) | (0.0702) | (0.473) |
| Observations | 56,736 | 56,736 | 56,736 | 56,736 | 56,736 | 56,736 |
|  |  |  |  |  |  |  |
| *Panel B: Early Education Spending* |  |  |  |  |  |  |
| Early education spending ($1,000 per child 0-18, 2016 dollars) | 0.612** | -0.484 | 0.492** | -0.199 | 0.521** | -0.600 |
|  | (0.137) | (0.538) | (0.142) | (0.337) | (0.136) | (0.407) |
| Individual covariates | Yes | Yes | Yes | Yes | Yes | Yes |
| Year FE | No | Yes | No | Yes | No | Yes |
| State covariates | No | Yes | No | Yes | No | Yes |
| State FE | No | Yes | No | Yes | No | Yes |
| Constant | -0.0641 | 2.120* | 0.0841+ | 1.674* | 0.0241 | 1.261* |
|  | (0.0491) | (0.885) | (0.0452) | (0.866) | (0.0554) | (0.514) |
| Observations | 56,736 | 56,736 | 56,736 | 56,736 | 56,736 | 56,736 |

Source/Notes: Authors’ analysis from 2003/2004, 2007/2008, and 2011/2012 Waves of the National Survey of Children’s Health, Urban Institute’s State-by-State Spending on Kids Dataset from 1998 to 2016, and National Institute for Early Education Research’s 2003, 2007, and 2011 State Preschool Yearbook. ** p<0.01, * p<0.05, + p<0.1

**References**

1. Pedlow S, Luke JV, Blumberg SJ. Multiple Imputation of Missing Household Poverty Level Values from the National Survey of Children with Special Health Care Needs, 2001, and the National Survey of Children’s Health, 2003 [Internet]. Washington D.C.: Centers for Disease Control and Prevention; 2007 Jun. Available from: https://www.cdc.gov/nchs/data/slaits/mimp01_03.pdf
